# Peripheral arterial disease in antiretroviral therapy naïve HIV infected patients – an experience from eastern India

**DOI:** 10.1186/1471-2334-14-S3-P50

**Published:** 2014-05-27

**Authors:** Dibbendhu Khanra, Arunansu Talukdar, Manjari Saha, Ashutosh Dey

**Affiliations:** 1 Department of General Medicine, Medical College and Hospital, Kolkata, India; 2 Department of Radiodiagnosis, Medical College and Hospital, Kolkata, India

## Background

HIV induced endothelial dysfunction leads to premature atherosclerosis which may manifest as peripheral arterial disease (PAD) of lower limbs. Studies are not available on prevalence of PAD among antiretroviral therapy (ART) naïve HIV infected population. Our objective was to explore the prevalence of PAD among HIV seropositive cases, and to determine relation of PAD with HIV infectivity and its correlation with CD4 count.

## Methods

This hospital based cross sectional study included 100 consecutive newly diagnosed HIV seropositive ART naïve cases (age 20-49 years) and 100 age and sex matched HIV seronegative controls. PAD was diagnosed by Doppler study of lower limb arteries.

## Result

Prevalence of PAD was significantly more among HIV infected cases (71%) in comparison to HIV negative controls (p <0.001). Among the cases of PAD, 53% (38/71) patients were asymptomatic. Active tuberculosis was present in 25% (18/71) of HIV infected PAD patients. The mean CD4 count among the HIV positive PAD cases was 220 cells/ dl. HIV sero-positivity was found to be independently contributing to development of PAD. Male gender, concurrent tuberculosis and low CD4 count came out to be individually contributing to PAD among HIV sero-positive cases in multivariate analysis.

## Conclusion

Prevalence of symptomatic and asymptomatic PAD was high in the ART naïve HIV-infected cases of relatively younger age group. HIV infected patients should undergo evaluation for lower limb peripheral arterial diseases in appropriate background. Larger population based prospective study involving HIV seropositive patients would enlighten the temporal association of PAD and other cardiovascular morbidities.

